# Pulmonary Infection Caused by *Mycobacterium malmoense* in a Chronic HIV-Infected Individual Participating in a Therapeutic Vaccine Trial

**DOI:** 10.3390/vaccines9101103

**Published:** 2021-09-29

**Authors:** Joana Silva Marques, Marta Bodro, Berta Torres, Felipe García, José Antonio Martínez, Lorna Leal

**Affiliations:** 1 Infectious Diseases Department, Hospital Clinic Barcelona, University of Barcelona, 08036 Barcelona, Spain; 6814@hstviseu.min-saude.pt (J.S.M.); mbodro@clinic.cat (M.B.); btorres@clinic.cat (B.T.); felipegarcia@ub.edu (F.G.); jamarti@clinic.cat (J.A.M.); 2 Internal Medicine Department, Centro Hospitalar Tondela-Viseu, 3504-509 Viseu, Portugal

**Keywords:** *Mycobacterium malmoense*, HIV, therapeutic vaccine

## Abstract

We report a case of *Mycobacterium malmoense* pulmonary infection and HIV-1 chronic co-infection in a 60-year-old man while participating in an HIV-1 therapeutic vaccine clinical trial and during the analytical treatment interruption. We present clinical and therapeutic features of a complicated *M. malmoense* pulmonary infection along with discussion of the possible relation to the HIV-1 cure-related interventions.

## 1. Introduction

*Mycobacterium malmoense* is a slow-growing non-tuberculous mycobacterium (NTM) that was first isolated in 1954 in Malmö, Sweden. Clinical cases have been extensively described in north and north western Europe [1,2] but infections in other areas are still rare. The most frequently associated clinical syndromes of *M. malmoense* infection include pediatric isolated lymphadenitis and adult tuberculous-like pulmonary infection. To date, very few cases of *M. malmoense* have been reported in Spain and only one case in an individual that was co-infected with HIV-1 [3].

HIV-1 antiretroviral treatment (ART) has improved life expectancy and quality of life in people living with HIV-1, but it is a lifetime treatment unable to cure or eradicate the infection. Therefore, finding new therapeutic strategies has become a priority, and the most promising ones are vaccines [4].

In a tertiary center in Barcelona, we conducted a clinical trial evaluating a dendritic cell-based (DC) vaccine in combination with interferon alpha 2a (IFNα2a) followed by a 12-week analytical treatment interruption (ATI) (NCT02767193). We describe a case of *M. malmoense* pulmonary infection in an HIV-1 co-infected man living in Barcelona and participating in the referred trial.

## 2. Clinical Case

The patient is a 60-year-old Swedish male and chronic smoker. He was diagnosed with an HIV-1 infection in February 2008 with 752 cells/mm^3^ CD4 T-cell count and 4.53log^10^ viral load (VL). The chest X-ray was normal and mantoux test negative. A year later antiretroviral treatment was started. Since then, plasma VL has been suppressed and CD4 T-cell count > 500 cells/mm^3^.

In May 2016 he was enrolled in an HIV therapeutic vaccine clinical trial. For vaccine production we needed autologous virus, therefore the patient had to stop ART after enrollment and during 9 weeks then ART was restarted. Although plasma VL increased up to 4.2log^10^, the CD4 T-cell count maintained >500 cells/mm^3^ and the patient remained asymptomatic. Ten months after restarting ART he started vaccination and received two doses of DC vaccine bi-weekly for 6 weeks. Vaccine was well tolerated and no complications were reported. The same day he received the last dose of DC vaccine, ART was interrupted and continued study treatment with pegylated INFα2a in a subcutaneous 180 mcg dose every week for 3 weeks. The patient referred to having chills, asthenia, myalgias and fever after each of the first two doses of INFα2a. Since symptoms had been described as related to interferon-based treatments, no further assessment was conducted.

**Figure 1:**
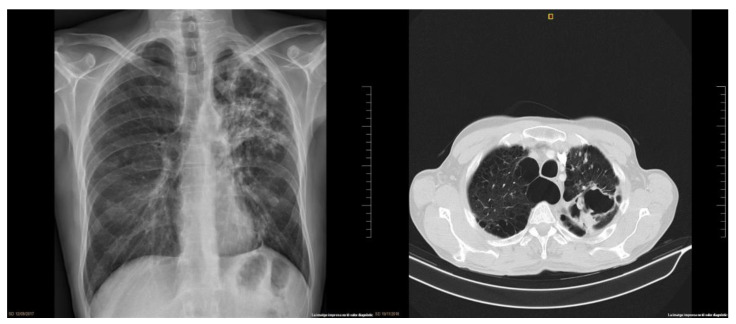
Lung images: (1) lung chest X-ray at diagnosis shows a left apical cavitation and surrounding infiltrate; (2) lung chest CT scan after clinical impairment shows central emphysema, fibrosis in both upper lobes with significant loss of volume, apical left lobe and diffuse bronchiectasis and a large apical cavity in left lung of approximately 7.1 cm.

After 1 week since the last dose of INFα2a and 3 weeks after ATI, the patient’s symptoms worsened and he also had productive cough and dyspnea. A chest X-ray was performed (Figure 1) and showed a left apical nodular image that suggested a granuloma. A sputum smear contained numerous acid-fast bacilli, and a nucleic acid amplification testing (GeneXpert) discarded *Mycobacterium tuberculosis* so a NTM infection was confirmed. Antimicrobial therapy was started with levofloxacin 750 mg, azithromycin 500 mg, rifampin 600 mg and ethambutol 1200 mg daily. At this moment the CD4 T-cell count was 787 cells/mm^3^ and VL 3.9log^10^ copies/mL. Due to the patient’s preference, ART was restarted until 2 weeks later. A Matrix-assisted laser desorption/ionization time-of-flight mass spectrometry (MALDI-TOF-MS) was used to characterize the *Mycobacterium* as *malmoense* and whole genome sequencing confirmed the results. The culture was positive and isolates were identified as *Mycobacterium malmoense.* Although the patient was originally from Sweden, he had been living in Spain for more than 15 years, so detailed epidemiologic and potential environmental exposure was assessed and revealed him travelling and working at least each spring in Sweden. After 16 weeks since starting antimicrobial therapy, we decided to continue with a three-drug regimen and stopped ETB. Treatment prescriptions and modifications are presented in Table 1. At this moment the blood test showed no abnormalities, CD4 T-cell count > 500 cells/mm^3^ and undetectable VL.

The antibiogram was obtained after approximately 18 weeks and the initial treatment was adjusted (Table 1). Due to several side-effects and different susceptibilities in following antibiograms, antimicrobial treatment suffered several changes that can be followed in Table 1. In March 2018, 6 months after the first *M. malmoense* isolation, the culture was negative and it was decided to continue this treatment for 12 more months.

In November 2018, patient came to the clinic referring non-life-threatening hemoptysis. We suspected therapy failure and adjusted treatment (Table 1). A chest computed-tomography (CT) was performed and showed central emphysema, fibrosis in both upper lobes with significant loss of volume in apical left lobe and diffuse bronchiectasis and a large apical cavity in the left lung (Figure 1). A respiratory endoscopy was performed and it did not find any endobronchial lesion. All the tests performed discarded active infections or malignancies. In May 2019, after 20 months under continuous antimicrobial therapy, and repeatedly negative cultures for 14 months, the treatment was interrupted. The patient continued ART as usual.

During subsequent follow-up the patient referred to intermittent mild hemoptysis and, considering the chest CT findings, we proposed surgical intervention. In January 2020 he underwent a left lung upper successful lobectomy. Pathology described acid-fast bacilli microorganisms within multiple necrosating granulomas. Unluckily, no culture was performed. With these results, a persistent mycobacterial infection could not be discarded. We decided to continue the assessments with positron emission tomography (PET) and restarted the antimicrobial regimen. Initially, PET reported pathological uptake of fluor-deoxi-glucose (FDG) in various nodular lesions on the left lung parenchyma and several thoracic lymphadenopathy that was interpreted as related to mycobacterial infection. Later images showed a gradual and significant improvement, so after 9 months treatment was interrupted.

To date patient is in good health, asymptomatic and continues his follow-up at an HIV-clinic.

## 3. Discussion

To the best of our knowledge, we describe the first reported case of *M. malmoense* infection during supervised antiretroviral treatment interruption in a chronic HIV-1 co-infected patient participating in an HIV-1 therapeutic vaccine clinical trial. The most frequent disease presentation is pulmonary infection that resembles mycobacterium tuberculosis (TBC) [5] and pre-existing pulmonary diseases, especially obstructive pulmonary disease, are a risk factor for pulmonary infection with *M. malmoense* [6].Our patient is a chronic smoker and a chest CT showed previous lung damage.

Treatment for NTM is long-lasting and it should be maintained for a minimum of 12 months after culture conversion. The British Thoracic Society (BTS) guidelines recommend treating *M. malmoense* pulmonary disease with rifampin, ethambutol and a macrolide and if severe, to add an injectable aminoglycoside [7]. These recommendations are based on the main findings of two randomized clinical trials [8,9] but both studies excluded HIV-infected individuals. Our patient started antimicrobial treatment with a four-drug regimen following NTM treatment recommendations in HIV-infected individuals without ART [10]. During his follow-up, we made some changes because of intolerance (see Table 1). Our patient still had positive smear and culture after 5 months of first-line recommended treatment, therefore we decided to take into consideration the susceptibility test for prescription even though there are controversies about reliability [9]. The patient was participating in a DC based HIV therapeutic vaccine combined with IFNα2a clinical trial that required ART interruption twice within a 15-month period. DC based immunotherapy has been widely studied in different scenarios such as cancer and infectious diseases including HIV-1, and has demonstrated a good safety profile [11,12]. Interferon alpha is a cytokine with a useful infection-control effect, but its exact mechanisms of action are still unclear. Th1 type cellular activity plays a key role in the control of mycobacterium and IFN plays a role in the regulation of Th1 responses [13], and thus has been proposed as a therapeutic agent in mycobacterial infections with controversial results. Giosuè and Cols studied the effects of aerosolized IFNα in TBC as part of conventional chemotherapy and found a better and faster sputum conversion and clinical improvement in patients that received the combined therapy [14]. This same strategy has been used in multi-drug resistant TBC with encouraging results [15]. In contrast, a few cases of TBC reactivation and exacerbation during a prolonged IFNα therapy for viral hepatitis have been described [16,17]. No clinical studies of IFN-based therapies and NTM have been conducted, but animal models have shown also different effects [18]. There is no uniform picture yet as to the function of type 1 IFN in mycobacterial infections and we cannot discard that this therapy might have an implication in our patient’s *M. malmoense* reactivation and outcome.

Except for a few cases describing non-supervised ART interruption and retroviral rebound syndrome [19,20] with different outcomes, other larger studies with even longer interruptions did not report any significant side effects [21,22], but the negative interaction between HIV and mycobacterium due to impaired cellular immunity has been described; hence it is possible that ATI favors bacterial reactivation.

## 4. Conclusions

We described an *M. malmoense* pulmonary infection in an HIV co-infected individual with a torpid outcome, and although this mycobacterium is highly pathogenic and difficult to treat, we cannot discard the effect of our interventions in the reactivation and evolution of this infection.

Particular attention should be paid to latent infections, beyond TBC, when using immunotherapy. Routine chest X-ray has not been implemented in HIV therapeutic vaccine clinical trials; our case report emphasizes the need to implement this evaluation, particularly before ATI.

## Figures and Tables

**Table 1:** Treatment prescriptions and modifications.

	2017	2019							2021	
Weeks	0–4	16	22	26	39	41	56	80	117 (January)	125 (March)
ANTIMYCOBACTERIAL TREATMENT (MCBT)	LVX 750 mg AZM 500 mg RIF 600 mg ETB 1200 mg	LVX 750 mg AZM 500 mg RIF 600 mg	AZM 500 mg ETB 1200 mg PRTN 1000 mg	AZM 500 mg ETB 1200 mg PRTN 750 mg	AZM 500 mg ETB 1200 mg MFX 400 mg LZD 600 mg	AZM 500 mg ETB 1200 mg MFX 400 mg	AZM 500 mg ETB 1200 mg MFX 400 mg RFB 300 mg AMK 1grm IV			AZM 500 mg ETB 1200 mg MFX 400 mg RFB 300 mg
INTERVENTION	Week 4 → Resume ART ABC 600 mg 3TC 300 mg DTG 100 mg	Worsened dizziness → Stop ETB	Following antibiogram → Stop LVX and RIF Restart ETB Start PRTN Reduce DTG	Reduce PRTN Ophtalmology OK	Following antibiogram → Stop PRTN Start MFX and LZD	Restart MFX	Start RFB AMK TX 18 days Endoscopy BAL no malignancies	After 14m STOP MCBT Continue ART	Left apical lobectomy	Restart MCBT STOP in JAN 2021 Total duration 112w
MICROBIOLOGY (−) negative (+) positive	QUAN (−) Sputum (+) Gene-xpert (−) Culture (+) Maldi-Tof Complete genome sequencing	Sputum (−) Culture (+)	Sputum (−) Culture (−)	Sputum (−) Culture (−)		Sputum (−) Culture (−)	Sputum (+) Culture (−) BAL: Aspergillus (−) Respiratory virus (−) P. Carinni (−) Smear (−) Culture (−)	Sputum (−) Culture (−)		Sputum (−) Culture (−)

MCBT: mycobacterial treatment; LVX: levofloxacin; AZM: azithromycin; RIF: rifampin; ETB: ethambutol; PRTN: protionamide; AMK: amikacin;; LZD: linezolid; MFX: moxifloxacin; RFB: rifabutin; ABC: abacavir; 3TC: lamivudine; DTG: dolutegravir; ART: antiretroviral treatment; QUAN: quantiferon; BAL: bronchoalveolar lavage;; TX: treatment; JAN: January.

## Data Availability

The data presented in this study are available on request from the corresponding author.
